# Glutaraldehyde-crosslinked *Rhizopus oryzae* whole cells show improved catalytic performance in alkene epoxidation

**DOI:** 10.1186/s12934-023-02026-0

**Published:** 2023-02-22

**Authors:** Lili Xu, Yimin Qin, Yufeng Song, Aixing Tang, Youyan Liu

**Affiliations:** 1 Medical College, Guangxi University, Nanning, 530004 China; 2 College of Marine Sciences, Beibu Gulf University, Qinzhou, 535011 China; 3 School of Chemistry and Chemical Engineering, Guangxi University, Nanning, 530004 China; 4 Key Laboratory of Guangxi Biorefinery, Guangxi University, Nanning, 530004 China

**Keywords:** Alkenes epoxidation, Lipase, glutaraldehyde-crosslinked whole-cells, Hydrogen peroxide, Na_3_C_6_H_5_O_7_

## Abstract

**Background:**

Existing methods for alkene epoxidation are based on lipase-catalysed perhydrolysis. However, the inactivation of the expensive lipase enzyme is problematic for enzymatic epoxidation at large scales due to the use of hydrogen peroxide and peracids at high concentrations in the reaction. The immobilisation of whole cells appears to be a promising approach to alleviate this problem.

**Results:**

A green oxidation system containing hydrogen peroxide, Na_3_C_6_H_5_O_7_, an acyl donor, and glutaraldehyde (GA)-crosslinked cells of *Rhizopus oryzae* was developed for the epoxidation of alkenes. GA-crosslinked cells of *Rhizopus oryzae* were adopted as a biocatalyst into the epoxidation system. A variety of alkenes were oxidised with this system, with a 56–95% analytical yield of the corresponding epoxides. The catalytic performance of the crosslinked treated cells was substantially improved compared to that of the untreated cells and the initial reaction rate increased from 126.71 to 234.72 mmol/L/h, retaining 83% yields even after four batches of reactions. The addition of 3.5 mmol Na_3_C_6_H_5_O_7_ not only acts as an acid-trapping reagent to eliminate the negative effect of the carboxylic acid on the alkene oxide but also forms a saturated salt solution with the aqueous phase, affecting the concentration of H_2_O_2_ in the three phases and thus the epoxidation reaction. Organic solvents with a logP value > 0.68 were good at producing hydroxy peracids; however, this method is only suitable for oxidation in a two-liquid phase.

**Conclusions:**

Compared with other lipase biocatalysts, the GA-crosslinked whole-cell biocatalyst is inexpensive, readily available, and highly stable. Therefore, it can be considered promising for industrial applications.

**Supplementary Information:**

The online version contains supplementary material available at 10.1186/s12934-023-02026-0.

## Introduction

Epoxides play an important role in organic synthesis and are widely used as intermediates for the synthesis of various high-value compounds [1, 2]. An alkene epoxidation reaction is an important approach for obtaining the epoxy compound. Until now, various epoxidation reaction modes have been reported; based on the catalyst, these include metal catalytic epoxidation [3], Prileshajev epoxidation [4], enzyme cyclooxidation [5–8], and chemoenzymatic oxidation [9]. Because biocatalysts have the characteristics of mild reaction, high selectivity, and high purity, the biotransformation processes continually attract interest.

In terms of chemoenzymatic epoxidation, it was established that the Prileshajev epoxidation reaction was helped along by an enzyme, whereby organic acid and hydrogen peroxide (H_2_O_2_) as substrates were used in the synthesis of peroxy acid in situ. When alkene is present in the system, peroxynitric acid immediately transfers oxygen to the C=C bond to form epoxy. Compared to cyclooxidation mediated by monooxygenases, chemoenzymatic epoxidation does not require cofactors and has a good organic solvent tolerance, allowing the use of non-haem-type peroxidases [10] or lipases [11]. Because lipases have certain advantages in pricing and thermal stability, many successful examples of alkenes epoxidation have been reported, including the synthesis of aliphatic alkene, cyclene, and vinyl aromatic compounds [12, 13]. Although lipases have been isolated from various microbial genera, including *Candida antarctica*, *Humicola *sp., *Pseudomonas *sp., *C. cylindracea*, *Aspergillus niger*, *Rhizopus chinensis*, *R. oryzae*, and *Rhizomucor mieheii* [14–16]*,* the most widely used lipases are *C. antarctica* lipase B (CALB) [15, 17]. This is due to its higher catalytic activity for "perhydrolysis" [18], that is, acting as the catalyst for chemoenzymatic epoxidation. The lipases isolated from the other had low reactivity [19].

The limited stability of lipases due to highly-concentrated H_2_O_2_ and organic solvents in the reaction system represents another bottleneck. Therefore, the lipases need to be immobilised rather than existing in a free state. Besides the widely used Novozym435, an immobilised CALB [20–22], various immobilisation methods have been tried to improve the stability of lipases. Tzialla et al. showed that immobilisation of CALB on an organic-modified mondolite nano clay retained up to 90% of the initial activity of CALB. However, long-term stability was not desirable, retaining only about 60% of the initial activity after four reaction cycles [23]. Tudorache et al. achieved long-term stability for α-pinene epoxidation using either crosslinked lipase CALB or lipase CALB with calcium brown algae as the carrier; unfortunately, the selectivity of α-pinene epoxide was low [24, 25].

According to these reports, enzyme source and operation stability remain a bottleneck in the development of a chemoenzymatic epoxidation process for industrial application. The *R. oryzae* lipase (ROL) was used to perform chemoenzymatic oxidation to caryophyllene to form a mono-epoxide product, with a conversion rate of 16% [26]. Here, it was shown that ROL should theoretically possess a catalysing function for the perhydrolysis reaction. Based on the above, we selected *R. oryzae* CGMCC 3.5040 as the enzyme-production strain for alkene epoxy catalysis. We found that *R. oryzae* CGMCC 3.5040 whole cells can catalyse the α-pinene epoxidation reaction, but with low catalytic activity and operational stability. Therefore, we tried to immobilise the whole cells. Upon evaluation, glutaraldehyde (GA) crosslinking was selected to strengthen whole cells because it can play a supporting and fixing role [27]. Sena et al. used a recombinant *Pichia pastoris* strain to express lipase from *R. miehei* (RML) on the cell surface as a new biocatalyst for biodiesel production from residual agro-industrial fatty acids. There, it was revealed that treatment using GA-immobilised RML-PIR1_ increased the stability and reusability of the biocatalyst. The RML-PIR1-GA scaffold retained 87.9% of its initial activity after six uses, whilst the activity of unmodified RML-PIR decreased by 40% after the first use [28]. We found that by using GA-crosslinking treatment on *Rhizopus oryzae*, the whole-cell catalyst could improve the catalytic activity and expressed rather good operational stability compared to those of untreated cells in the chemoenzymatic epoxidation system.

After the construction and immobilisation of the GA *R. oryzae* whole-cell, epoxidation efficiency was investigated. α-Pinene, octene, and styrene were selected as three representative substrates for systematic analysis of whole-cell catalytic alkene epoxidation. These methods offer useful applications for synthetic modifications and scalable green processes.

## Materials

### Microorganisms and materials

The lipase-producing fungal strain CGMCC 3.5040 was obtained from China General Microbiological Culture Collection Center (CGMCC; Beijing, China). Norbornene oxide was purchased from Sigma-Aldrich (Shanghai, China). Octane, octane oxide, 1-hexene, 1-hexene oxide, α-pinene, α-pinene oxide, norbornene, cyclohexene, cyclohexene oxide, styrene, styrene oxide, α-methylstyrene, α-methylstyrene oxide, octanoic acid, ethyl acetate, hexane, acetic acid, lauric acid, acetonitrile, sodium carbonate (Na_2_CO_3_), disodium hydrogen phosphate (Na_2_HPO_4_), and trisodium citrate dihydrate (Na_3_C_6_H_5_O_7_), soybean oil were purchased from Aladdin Chemistry Co., Ltd (Shanghai, China). Hydrogen peroxide (30%), DMSO, acetone, isopropanol, trichloromethane, and toluene were of analytical grade and purchased from Damao Chemical Reagent Factory (Tianjin, China). A 50% GA solution of analytical grade was obtained from Macklin (Shanghai, China). All of the remaining reagents used were of analytical grade.

### Whole-cell biocatalyst preparation

A culture medium was prepared by dissolving 30 g soybean oil, 70 g peptone, 1.0 g NaNO_3_, 1.0 g KH_2_PO_4_, and 0.5 g MgSO_4_·7H_2_O in 1 L distilled water. Flasks (500 mL) containing 100 mL of the basal medium were inoculated by aseptically transferring spores (± 10^6^ spores) from an agar slant and incubated for about 48 h at 30 °C on a reciprocal shaker (150 rpm). *Rhizopus oryzae* whole cells were separated from the culture broth by filtration and washed with tap water.

### Whole cell GA treatments

Cells were incubated in 0.1% GA solution (0.01 M phosphate buffer, pH 7.0) for 1 h. Cells without GA treatment were used as blanks after separation by filtration. The GA-treated cells were washed with phosphate buffer (0.01 M, pH 7.0) and frozen in a − 80 °C refrigerator for 30 min. Next, the cells were dried under a vacuum ± 20 h.

### Epoxidation with GA-crosslinked whole cells and untreated cells

α-Pinene (1 mmol), octanoic acid (1 mmol), 30% H_2_O_2_ (7.5 mmol), and trisodium citrate (3.5 mmol) in toluene (1.5 mL) were added to 200 mg GA-crosslinked whole cells or untreated cells. The mixture was shaken in a test tube at 180 rpm and 30 °C for 6 h. Next, the upper toluene solution of the reaction sample was aspirated and added to a toluene solution (1.5 mL) containing α-pinene (1 mmol) and octanoic acid (1 mmol) for a new batch of epoxidation. Samples were collected at different time points for a time course analysis of α-pinene epoxidation catalysis by GA-crosslinked whole cells and untreated cells. The concentrations of α-pinene and α-pinene oxide were determined by gas chromatography.

### Determining the effect of reaction factors on alkene epoxidation by whole cells

The straight alkene octene, the cycloalkene α-pinene, and the vinyl aromatic compound styrene were selected as model substrates for each alkene class and the effect of reaction factors were investigated on the three model substrates.

Six organic solvents were selected as solvents for α-pinene, octene, and styrene epoxidation. A mixture of α-pinene, octene, or styrene (1 mmol), octanoic acid (1 mmol), H_2_O_2_ (30% [v/v] aqueous solution, 7.5 mmol), trisodium citrate (3.5 mmol), and GA-crosslinked whole cells (200 mg) in different organic solvents (1.5 mL) was shaken at 180 rpm and 30 °C for different reaction times (α-pinene: 6 h, octene: 50 h, styrene: 58 h).

A mixture of α-pinene, octene, or styrene (1 mmol), acyl donor (1 mmol; acetic acid, octanoic acid, lauric acid, ethyl acetate), except in the control group, H_2_O_2_ (30% [v/v] aqueous solution, 7.5 mmol), trisodium citrate (3.5 mmol), and GA-crosslinked whole cells (200 mg) in toluene (1.5 mL) was shaken at 180 rpm and 30 °C for different reaction times (α-pinene: 6 h, octene: 50 h, styrene: 58 h).

To a solution of α-pinene, octene, or styrene (1 mmol), octanoic acid (1 mmol), 30% (v/v) H_2_O_2_ (1−10 mmol), and trisodium citrate (3.5 mmol) in toluene (1.5 mL) was added to GA-crosslinked whole cells (200 mg). Then, the mixture was shaken at 180 rpm and 30 °C for different reaction times (α-pinene: 6 h, octene: 50 h, styrene: 58 h).

A 1.5 mL toluene solution of α-pinene, octene, or styrene (1 mmol), octanoic acid (1 mmol), and H_2_O_2_ (30% [v/v] aqueous solution, 7.5 mmol) was prepared. Next, the solution was combined with 1−5 mmol acid-trapping reagents (Na_2_CO_3_, Na_2_HPO_4_, and Na_3_C_6_H_5_O_7_), except in the control group, and 200 mg of GA-crosslinked whole cells were added. The salts did not completely dissolve in the solution, with a fraction remaining in the solid phase. The mixed solvents were shaken at 180 rpm and 30 °C for different reaction times (α-pinene: 6 h, octene: 50 h, styrene: 58 h).

The data at 30 min were used to measure the initial reaction rate for α-pinene. The data between 30 and 60 min were used to measure the initial reaction rates for octene and styrene. Groups with inactivated cells were used as the blank groups. All experiments were performed in triplicate.

### General procedure for the epoxidation of olefins

A solution of the alkene (1 mmol) and octanoic acid (1 mmol) in toluene (1.5 mL) was added to a solution containing H_2_O_2_ (30% [v/v] aqueous solution, 7.5 mmol), Na_3_C_6_H_5_O_7_ (3.5 mmol), and *R. oryzae* whole cells (200 mg). The mixture was shaken at 180 rpm and 30 °C for different reaction times. Samples were collected at different time points.

### Analytical methods

The reaction mixture was centrifuged and filtered using a 0.22 µm filter head. The supernatant was analysed using a gas chromatography machine (GC-2010 Plus, Shimadzu, Tokyo, Japan) with flame ionisation detection (GC-FID) on a Rxi-5Sil (Shimadzu, 30 m × 0.32 mm; ID × 0.25 µm). The temperatures of the injection room and detector were 250 °C and 280 °C, respectively. The column temperature was set to 80 °C for 3 min and then increased to 160 °C at a rate of 20 °C/min, and then held for 1 min. The identity of the product (α-pinene oxide) was confirmed by comparison with a standard sample. For other alkenes and the corresponding epoxides, the temperature profile was: 50 °C for 3 min and then increased to 160 °C at a rate of 10 °C/min and then held for 1 min. The identity of the product (alkene oxide) was confirmed by comparison with an authentic sample.

Scanning electron microscopy (SEM) observations of the mycelium, before and after the GA-crosslinking treatment, as well as the subsequent forms in the presence or absence of buffer salts in the epoxidation reaction, were done using a Tescan MIRA LMS field-emission SEM (Tescan Ltd., Brno, Czech).

### H_2_O_2_ concentration determination

Here, Ce(SO_4_)_2_ was reacted with H_2_O_2_ according to the following reaction: 2Ce^4+^  + H_2_O_2_ = 2Ce^3+^  + 2H^+^  + O_2_. To this end, Ce(SO_4_)_2_ was dissolved in 0.5 mol/L sulfuric acid to a concentration of 0.1 mol/L, which was used as the working solution. Next, the sample was added to 5 mL of working solution and after 3 min of reaction, 200 µL of the mixture was measured at 480 nm using a microplate reader (BioTek). A standard curve was constructed to extrapolate the concentration of H_2_O_2_ in the samples.

## Results and discussion

### Epoxidation efficiency of GA-crosslinked whole cells versus untreated cells

**Fig. 1:**
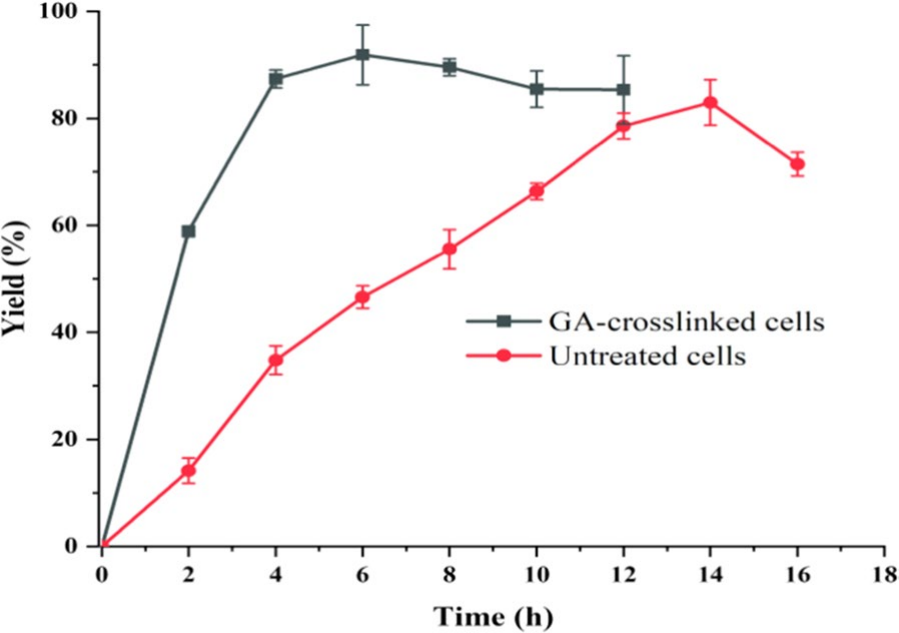
Evolution of the epoxidation reaction over time with GA-crosslinked whole cells and untreated cells

Glutaraldehyde-crosslinking is a simple, easy, and effective way to immobilize cells [29–31]. As seen in Table 1, the reusability of untreated cells was poor. The conversion of untreated cell catalyst decreased from 81 to 56% on the second reuse, with only 22% remaining on the third reuse, and only 2% yield remaining. This suggests that even for whole cells, it is difficult to adapt to the presence of high concentrations of H_2_O_2_ and organic solvents in the epoxidation system. There were two significant improvements in the catalytic properties of the cells after GA-crosslinking. Firstly, the reaction efficiency of the cells increased from 81 to nearly 100% and the yield increased from 56 to 90%. Considering the time course for α-pinene epoxidation catalysed by GA-crosslinked whole cells and non-crosslinked cells (Fig. 1), the initial reaction rate of the crosslinked whole cells increased from 126.71 to 234.72 mmol/L/h in untreated cells; the conversion was completed in 6 h with a 91.88% yield. The non-crosslinked cells took 14 h to reach an 82.97% yield after equilibrium. Most surprisingly, the reusability of the GA-crosslinked whole cells was greatly improved, with the conversion rate remaining at 96% and the yield at 83% after four reuse cycles (Table 1, entry 2).

**Table 1:** Reusability of whole cell catalysts with GA-crosslinked whole cells and untreated cells

Entry	Cross-linking method	First conversion (%)	First yield (%)	Second conversion (%)	Second yield (%)	Third conversion (%)	Third yield (%)	Fourth conversion (%)	Fourth yield (%)
1	Untreated cells	81.59 ± 3.99	56.1 ± 1.98	55.35 ± 7.83	32.21 ± 5.82	22.2 ± 2.58	2.3 ± 0.96	–	–
2	GA cross-linked cells	99.76 ± 0.05	90.31 ± 1.45	99.16 ± 2.05	84.87 ± 3.45	99.32 ± 1.35	87.65 ± 2.31	96.12 ± 0.05	83.25 ± 2.31

Reaction conditions: a mixture of α-pinene (1 mmol), octanoic acid (1 mmol), GA-crosslinked whole cells or untreated cells (200 mg), H_2_O_2_ (30% [v/v] aqueous solution, 7.5 mmol), and Na_3_C_6_H_5_O_7_ (3.5 mmol) in toluene (1.5 mL) was shaken at 180 rpm and 30 °C for 6 h. Cell recycling: reaction sample was aspirated the upper toluene solution, and then a new toluene solution (1.5 mL) was added to the reaction tube for a new batch of epoxidation

The crosslinking of cells with glutaraldehyde is a conventional and effective strategy. Sun et al. found that the stability of the *R. oryzae* IFO4697 whole-cell catalyst for the reuse of methanolysis of oils and fats was significantly improved after GA-crosslinking [32]. Compared to this previous study, our results are more surprising, i.e., not only are the cells stable after crosslinking, but the reaction properties also change considerably. Bernhardt et al. reported that the substitution of Leucine in position 29 of perhydrolase with Proline (L29P), led to an increase in activity. The molecular basis for the increase in activity is the presence of a carbonyl group in the vicinity of the active site that stabilises H_2_O_2_ attack on a putative acyl-enzyme intermediate [33]. Glutaraldehyde-crosslinking could support and fix the cells, especially considering the membrane system (e.g., microtubules and endoplasmic reticulum) and the cell matrix and given that *Rhizopus oryzae* lipase is mainly located on the cell wall and cell membrane [34]. We speculate that the carbonyl group present in the GA molecule facilitates the formation of a stable tetrahedral conformation of H_2_O_2_, which facilitates the perhydrolysis reaction.

A long reaction time leads to the decomposition of the epoxide product and is not conducive to the industrial production of the product. Moreover, it also increases the toxicity of the organic solvent in the reaction system. Here, the whole-cell-catalysed epoxidation of α-pinene took ca. 6 h, which is similar to that required for Novozym 435 in the literature [12]. Crosslinking using a pH 7.0 GA solution was selected for further experimentation.

### GA-crosslinked whole-cell catalysis of the epoxidation of different olefins with H_2_O_2_ and octanoic acid

**Scheme 1:**
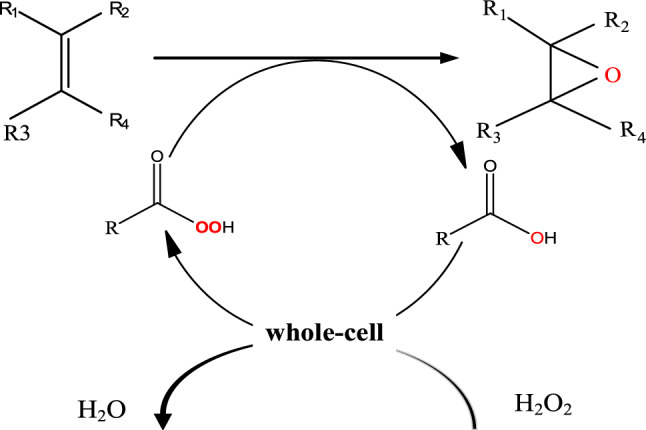
Epoxidation of alkenes through GA-crosslinked whole cells-catalyzed perhydrolysis of carboxylic acids

This new three-phase system (organic phase-solid phase-aqueous phase) was established using crosslinked whole cells for the epoxidation of alkenes. The substrate and products constitute the organic phase, H_2_O_2_ forms during the aqueous phase, and lipase cells constitute the solid phase, located in the upper organic phase (Scheme 1). Seven alkenes were selected for epoxidation; the straight chain alkenes were octene and hexene, the cyclic alkenes were α-pinene, norbornene, and cyclohexene, and the aromatic alkenes were styrene and α-methylstyrene. The identity of the product (alkene oxide) was confirmed by comparison with an authentic sample (Additional file 1: Fig. S1).

The order of the reaction equilibrium times for the alkenes are, α-pinene, borneolene, cyclohexene, α-methylstyrene, hexene, octene, and styrene (Table 2). According to the mechanism of the Prileshajev reaction (butterfly mechanism), the peroxide reagent is electrophilic while the alkene is nucleophilic. The rate of alkene epoxidation is significantly higher when the hydrogens on the alkenes are substituted with methyl substituents, which increase the electron density of the double bond, facilitating the Prileshajev reaction, so that the cyclic alkenes are the fastest in terms of epoxidation rate. α-Methylstyrene (Table 2, entry 7) also has a significantly faster epoxidation rate than styrene due to the presence of methyl substituents (Table 2, entry 6).

**Table 2:** Epoxidation of different alkenes

Entry	Alkene	Epoxide	Time/h	Yield (%)
1			50	56.24 ± 3.95
2			50	66.02 ± 2.77
3			6	92.04 ± 3.08
4			12	95.4 ± 2.7
5			20	95.15 ± 0.37
6			58	76.56 ± 1.22
7			35	92.27 ± 0.56

A mixture of alkene (1 mmol), octanoic acid (1 mmol), cell (200 mg), H_2_O_2_ (30% [v/v] aqueous solution, 7.5 mmol), and trisodium citrate (3.5 mmol) in toluene (1.5 mL) was shaken at 180 rpm and 30 °C

The cyclic alkenes and α-methylstyrene with high epoxidation rates had 92−95% yields, straight-linked alkenes with lower reaction rates (octene and hexene) had 56−66% yields, and styrene had 71% yields, indicating that higher catalytic activity is required for the epoxidation of straight-linked alkenes using lipases, thereby producing more peroxy acids.

The epoxidation reactions rates and yields of straight-linked alkenes were relatively slower and lower compared to cyclic and aromatic alkenes, respectively, which were consistent with the epoxidation of Novozym 435 [12]. Notably, the yields of α-pinene, norbornene, and cyclohexene (Table 2, entries 3–5) were all above 90%, indicating the potential of this method for practical application.

### Effects of reaction factors on the alkene epoxidation by GA-crosslinked whole cells

To investigate the key factors of the reaction system, the effects of organic solvents, acyl donors, H_2_O_2_ concentration, and various acid-trapping reagents on the epoxidation reaction systems were investigated using the straight alkene octene, the cycloalkene α-pinene, and the vinyl aromatic compound styrene as model substrates. According to preparatory experiments (Additional file 1: Fig. S2), the conversions and yields of α-pinene, octene, and styrene were measured after 6, 50, and 58 h of reaction, respectively.

#### Effect of organic solvents on alkene epoxidation

We investigated the effect of six organic solvents with different logP values on the epoxidation reaction of α-pinene, octene, and styrene (Table 3). The initial reaction rates of the three alkenes in chloroform, toluene, and n-hexane were all consistent with the pattern of their conversions and yields after reaching reaction equilibrium. The initial reaction rate in ethyl acetate was similar to that of the optimum solvent toluene, while its conversion and yield were the lowest among the four solvents, likely due to ethyl acetate acting as an acyl donor to form peroxy acids. The conversion and yield were lower due to the strong polarity. The three alkenes all had the highest initial reaction rate, with the best conversion and yield in toluene with a logP value of 2.5. However, the solvents with the lowest initial reaction rates for the three alkenes differed, with α-pinene and styrene having the lowest initial reaction rates in chloroform and octene having the lowest initial reaction rate in hexane.

**Table 3:** Alkene epoxidation in different organic solvents

Entry^a^	Substrate	Solvents	Log P	Initial reaction rate ^b^ (mmol/L/h)	conversion (%)^c^	yield (%)^c^
1		Acetonitrile	− 0.33	NA	NA	NA
2		Isopropanol	0.16	NA	NA	NA
3		Ethyl acetate	0.68	231.19 ± 9.56	41.42 ± 5.59	40.51 ± 2.84
4		Trichloromethane	2.0	32.55 ± 0.1	54.22 ± 2.55	45.33 ± 1.31
5		Toluene	2.5	232.88 ± 1.76	99.65 ± 0.07	92.04 ± 3.08
6		Hexane	3.50	206.99 ± 12.47	94.98 ± 5.49	90.78 ± 8.01
7		Acetonitrile	− 0.33	NA	NA	NA
8		Isopropanol	0.16	NA	NA	NA
9		Ethyl acetate	0.68	5.38 ± 1.44	18.22 ± 0.45	10.59 ± 0.63
10		Trichloromethane	2.0	2.55 ± 0.68	42.61 ± 4.53	19.17 ± 5.82
11		Toluene	2.5	5.79 ± 0.19	66.44 ± 0.91	56.24 ± 3.95
12		Hexane	3.50	1.93 ± 0.39	23.26 ± 2.14	18.33 ± 0.92
13		Acetonitrile	− 0.33	NA	NA	NA
14		Isopropanol	0.16	NA	NA	NA
15		Ethyl acetate	0.68	9.59 ± 0.45	13.36 ± 0.11	10.4 ± 0.82
16		Trichloromethane	2.0	6.64 ± 1.1	29.34 ± 0.41	27.72 ± 1.67
17		Toluene	2.5	9.59 ± 0.19	89.02 ± 0.64	76.56 ± 1.22
18		Hexane	3.50	7.19 ± 0.53	54.94 ± 0.32	53.19 ± 1.24

^a^A mixture of alkene (1 mmol), octanoic acid (1 mmol), cell (200 mg), H_2_O_2_ (30% [v/v] aqueous solution, 7.5 mmol), and Na_3_C_6_H_5_O_7_ (3.5 mmol) in different organic solvents (1.5 mL) was shaken at 180 rpm and 30 °C for different reaction times. ^b^The value obtained at 30 min for pinene and 60 min for octene and styrene, respectively. ^c^The value obtained at 6 h for pinene, 50 h for octene and 58 h for styrene, respectively

The polarity of the solvent had a strong effect on the reaction system, with the three alkenes all failing to undergo epoxidation in the organic solvents acetonitrile and isopropanol with logP values < 0.16. The moderately polar solvent toluene (logP 2.5) was more suitable as a solvent for this reaction system. The initial reaction speed of α-pinene in toluene (logP 2.5) was 232.88 mmol/L/h, greater than that of chloroform (logP 2.0) at 32.55 mmol/L/h and hexane (logP 3.5) at 206.99 mmol/L/h. Octene and styrene had smaller initial reaction rates relative to α-pinene, with the largest initial reaction rates in toluene at 5.79 mmol/L/h and 9.59 mmol/L/h, and styrene also had a larger initial reaction rate of 7.19 mmo/L/h in hexane (logP 3.5), further suggesting that hydrophobic solvents are more suitable for the whole-cell catalytic system. Nikolova et al. found that whole-cell biocatalysts require more water than enzymes in their catalytic reactions and that a water-free system requires almost no water [35]. This may be related to the interfacial activation of lipases and the presence of the aqueous phase reducing the toxic effect of the substrate, products, and organic solvents on the cells.

The conversions and yields of α-pinene, octene, and styrene were measured after 6, 50, and 58 h of reaction, respectively. Although the initial reaction rates of octene and styrene were small, good conversions and yields were obtained for all three alkenes in toluene after reaching the reaction equilibrium. The optimum conversion and yield were 99.65% and 92.04% for α-pinene, 66.44% and 56.24% for octene, and 89.02% and 76.56% for styrene, respectively. Tzialla et al. selected six solvents with a LogP ranging from − 0.33 to 4.8, in their study of immobilised enzyme-catalysed α-pinene epoxidation, and found that toluene with a logP value of 2.5 performed the best [23]. Ranganathan et al. also identified toluene as the best solvent among eight solvents during the optimisation of enzyme-catalysed monoterpene epoxidation using Taguchi's experiment [36]. Our experiment shows that toluene has excellent performance as a solvent for whole-cell catalytic epoxidation systems.

#### Effects of acyl donors on alkene epoxidation

To investigate the relationship between the ability of *R. oryzae* whole-cell lipases to catalyse the epoxidation of alkenes and the type of acyl donor, three straight-chain organic acids, acetic acid, n-octanoic acid, lauric acid, and ethyl acetate were used as acyl donors (Table 4). No acyl donor was added to the system to investigate the effect of the whole cells themselves on the reaction system. The initial reaction rates of the three alkenes in acetic acid, n-octanoic acid, lauric acid, and no acyl donor systems were all consistent with their conversion and yield at equilibrium. However, the initial reaction rates of α-pinene and styrene were greater than those of the no acyl donor system when ethyl acetate was used as the acyl donor. However, their conversions and yields were lower than those of the no acyl donor system. The three alkenes had minimum initial reaction rates and yields when acetic acid (C:2) was used as the acyl donor. Moreover, the three alkenes had optimum initial reaction rates and yields when n-octanoic acid (C:8) was used as the acyl donor.

**Table 4:** Alkene epoxidation with different types of acyl donors

Entry^a^	Substrate	Acyl donor	Initial reaction rate^b^ (mmol/L/h)	Conversion (%)^c^	Yield (%)^c^
1		None	69.43 ± 2.06	76.3 ± 1.32	61.52 ± 1.26
2		Acetic acid	38.1 ± 1.49	38.15 ± 0.77	31.76 ± 1.35
3		Octanoic acid	232.88 ± 1.76	99.65 ± 0.07	92.04 ± 3.08
4		Lauric acid	130.1 ± 20.6	81.12 ± 2.56	70.52 ± 1.22
5		Ethyl acetate	95.44 ± 1.99	49.36 ± 0.17	49.16 ± 1.82
7		None	1.79 ± 0.31	29.69 ± 1.01	21.65 ± 0.37
8		Acetic acid	1.29 ± 0.49	13.77 ± 1.24	10.26 ± 1.21
9		Octanoic acid	5.79 ± 0.19	66.44 ± 0.91	56.24 ± 3.95
10		Lauric acid	4.45 ± 1.21	64.63 ± 2.01	53.14 ± 1.24
11		Ethyl acetate	2.19 ± 0.45	27.74 ± 0.42	24.73 ± 0.47
13		None	4.04 ± 0.24	47.26 ± 2.2	37.32 ± 0.41
14		Acetic acid	1.92 ± 0.22	15.81 ± 0.48	15.73 ± 0.48
15		Octanoic acid	9.59 ± 0.19	89.02 ± 0.64	76.56 ± 1.22
16		Lauric acid	7.56 ± 0.47	83.03 ± 1.68	58.52 ± 2.19
17		Ethyl acetate	6.41 ± 0.78	40.68 ± 0.34	27.97 ± 0.98

^a^A mixture of alkene (1 mmol), acyl donor (1 mmol; acetic acid, octanoic acid, lauric acid, ethyl acetate), cell (200 mg), H_2_O_2_(30% [v/v] aqueous solution, 7.5 mmol), and Na_3_C_6_H_5_O_7_ (3.5 mmol) in toluene (1.5 mL) was shaken at 180 rpm and 30 °C for different reaction time

^b^The value obtained at 30 min for pinene and 60 min for octene and styrene, respectively

^c^The value obtained at 6 h for pinene, 50 h for octene, and 58 h for styrene, respectively

The type of organic acid has an important influence on the reaction, with medium carbon chain organic acids being more suitable as acyl donors for this reaction system. The initial reaction rate, conversion, and yield (232.88 mmol/L/h, 99.65% and 92.04%) were much higher than those of acetic acid (38.1 mmol/L/h, 38.15% and 31.76%) when n-octanoic acid (C:8) was used as the acyl donor for α-pinene. Compared to short-chain acetic acid (C:2), the three alkenes showed higher initial reaction rates and yields using lauric acid (C:12) as the acyl donor, especially for octene, where the initial reaction rate, conversion, and yield using lauric acid (C:12) (4.45 mmol/L/h, 64.63%, 53.14%) were similar to those using n-octanoic acid (C:8) (5.79 mmol/L/h, 66.44%, 56.24%) as the acyl donor. The combination of the initial reaction rates, conversions, and yields of the five different acyl donors suggests that more organic acids can be produced with medium carbon chains, which is also consistent with literature reports [9, 37].

Notably, the conversions and yields were 76.3% and 61.52% for α-pinene, 29.69% and 21.65% for octene, and 47.26% and 37.32% for styrene, respectively, when the system was not supplemented with organic acids alone. This suggests the presence of substances in the cells that can act as acyl donors to produce peracids catalysed by lipase. It has been noted that in the preparation of *R. oryzae* whole cells, soybean oil was added to the medium as a carbon source and lipase inducer and the cells adsorbed fatty acids onto the cell membrane as they grew [38].

#### Effects of H_2_O_2_ concentration on alkene epoxidation

In chem-enzyme epoxidation, H_2_O_2_ has an important influence on the reaction. This experiment investigated the effect of different contents of H_2_O_2_ on the reaction system (Table 5). When the H_2_O_2_ content was increased from 1 to 10 nmol, the initial reaction rate of all three alkenes gradually increased. Moreover, the conversions and yields of α-pinene and styrene both gradually increased and then decreased, while the conversion and yield of octene gradually increased and then remained stable. This indicates that the optimum H_2_O_2_ dosage differs for the three alkenes, with 5 nmol for α-pinene and 7.5 nmol for styrene and octene, which require more peroxy acids for epoxidation.

**Table 5:** Alkene epoxidation with different H_2_O_2_ content

Entry^a^	Substrate	Content of H_2_O_2_ (mmol)	Initial reaction rate^b^ (mmol/L/h)	Conversion (%)^c^	yield (%)^c^
1		1	93.99 ± 6.41	57.21 ± 0.82	49.17 ± 1.22
2		2.5	121.87 ± 9.26	91.99 ± 0.04	74.5 ± 1.99
3		5	176.15 ± 1.78	99.89 ± 0.08	91.37 ± 1.94
4		7.5	232.88 ± 1.76	99.65 ± 0.07	92.04 ± 3.08
5		10	239.81 ± 5.23	99.73 ± 0.11	86.75 ± 0.24
6		15	–	74.53 ± 5.6	67.96 ± 8.45
7		30	–	20.56 ± 7.12	12.8 ± 1.99
8		45	–	6.37 ± 5.28	6.25 ± 0.89
9		1	4.22 ± 0.09	22.17 ± 0.35	8.13 ± 0.54
10		2.5	4.35 ± 0.91	49.15 ± 0.36	36.68 ± 1.97
11		5	5.29 ± 0.05	62.11 ± 0.33	45.77 ± 2.3
12		7.5	5.79 ± 0.19	66.44 ± 0.91	56.24 ± 3.95
13		10	6.34 ± 2.35	63.95 ± 1.41	56.3 ± 0.59
14		1	6.31 ± 0.3	15.91 ± 0.88	14.25 ± 2.1
15		2.5	6.62 ± 1.18	68.82 ± 0.42	50.02 ± 3.94
16		5	9.23 ± 2.65	83.53 ± 0.42	65.35 ± 4.49
17		7.5	9.59 ± 0.19	89.02 ± 0.64	76.56 ± 1.22
18		10	9.72 ± 1.39	84.79 ± 1.15	70.38 ± 1.69

^a^A mixture of alkene (1 mmol), octanoic acid (1 mmol), 30% (v/v) H_2_O_2_ (1−10 mmol), and Na_3_C_6_H_5_O_7_ (3.5 mmol) in toluene (1.5 mL) was shaken at 180 rpm and 30 °C

^b^The value obtained at 30 min for pinene and 60 min for octene and styrene, respectively

^c^The value obtained at 6 h for pinene, 50 h for octene and 58 h for styrene, respectively

The H_2_O_2_ concentration was the key parameter affecting enzyme activity. The epoxidation yield of α-pinene was increased from 49.17 to 91.37% when the H_2_O_2_ content was increased from 1 to 5 nmol, but its yield decreased from 92.04 to 86.75%, when it was increased from 7.5 to 10 nmol. Moreover, the epoxidation yield of α-pinene decreased from 86.75 to 6.25% when the H_2_O_2_ content was further increased to 45 nmol. When the H_2_O_2_ content was increased from 1 to 10 nmol, the yield of octene epoxidation increased from 8.13 to 56.24% and then remained stable. The yield of styrene epoxidation increased from 14.25 to 76.56% and then decreased to 70.38%, indicating that at a low H_2_O_2_ dosage, the perhydrolysis reaction was affected resulting in insufficient peracid production and thus low efficiency of the epoxidation reaction. At a high H_2_O_2_ dosage Zhao et al. [39] modelled and analysed the behaviour of H_2_O_2_ on the lipase (PCL) from *Penicillium camembertii* and elucidated the mechanism of H_2_O_2_-induced lipase inactivation. This was done by simulating and analysing the behaviour of H_2_O_2_ on PCL.

#### Effect of buffer salts on alkene epoxidation

To solve the problem of alkene epoxide ring opening due to the presence of carboxylic acids in the epoxidation reaction system, some basic compounds (i.e., acid-binding agents) were added to balance the hydrogen ions in the reaction system, improving the selectivity of the epoxidation reaction. Xu et al. found the addition of dipotassium hydrogen phosphate increased the yield of styrene epoxide by 31% when using lipase for the epoxidation of styrene [12]. Su et al. found that the yield of the α-pinene epoxidation reaction increased from 40.7 to 61% with the addition of trisodium citrate [13]. It shows that the addition of a strong base and weak acid cache salt inhibits epoxide ring opening.

Here, Na_2_CO_3_, Na_2_HPO_4_, and Na_3_C_6_H_5_O_7_ were added directly to the reaction system, and it was found that Na_2_CO_3_ was not suitable (Table 6, entry 2), mainly because excess Na_2_CO_3_ caused the decomposition of H_2_O_2_. Moreover, Na_2_HPO_4_ and Na_3_C_6_H_5_O_7_ had a positive effect on the yield (Table 6, entries 4–6), while Na_3_C_6_H_5_O_7_ was more suitable as the acid-trapping reagent for this reaction system. When Na_3_C_6_H_5_O_7_ was added in the range of 1−3.5 mmol, the yields of the three olefins gradually increased and reached the maximum at 3.5 mmol; the yields decreased afterwards because the addition of too much Na_3_C_6_H_5_O_7_ would cause the initial reaction system to be weakly basic, which was unfavourable to the formation of epoxy products, and the optimal addition of the acid-trapping reagent was chosen to be 3.5 mmol.

**Table 6:** Alkene epoxidation with varied buffer salts

Entry^a^	Substrate	Buffer salts	Amount of buffer salts (mmol)	Initial reaction rate^b^ (mmol/L/h)	Conversion (%)^c^	yield (%)^c^
1		None	–	–	26.13 ± 0.25	6.52 ± 0.88
2		Na_2_CO_3_	3.5	–	82.95 ± 2.19	NA
3		Na_2_HPO_4_	3.5	198.07 ± 4.02	68.09 ± 1.88	46.71 ± 1.18
4		Na_3_C_6_H_5_O_7_	1	44.46 ± 1.05	40.46 ± 2.14	31.56 ± 0.11
5		Na_3_C_6_H_5_O_7_	3.5	232.88 ± 1.76	99.65 ± 0.07	92.04 ± 3.08
6		Na_3_C_6_H_5_O_7_	5	145.05 ± 2.23	94.32 ± 1.12	83.69 ± 4.23
7		None	–	–	3.11 ± 0.58	2.23 ± 0.56
9		Na_2_HPO_4_	3.5	5.17 ± 0.42	19.31 ± 0.88	8.56 ± 0.08
10		Na_3_C_6_H_5_O_7_	1	4.66 ± 0.95	10.72 ± 1.44	5.98 ± 0.8
11		Na_3_C_6_H_5_O_7_	3.5	5.12 ± 0.43	66.44 ± 0.91	56.24 ± 3.95
12		Na_3_C_6_H_5_O_7_	5	6.54 ± 1.21	61.53 ± 0.55	56.61 ± 0.93
13		None	-	-	5.13 ± 1.31	2.65 ± 0.78
15		Na_2_HPO_4_	3.5	9.21 ± 1.96	15.43 ± 0.37	8.75 ± 0.76
16		Na_3_C_6_H_5_O_7_	1	7.28 ± 1.5	16.06 ± 0.43	9.36 ± 0.73
17		Na_3_C_6_H_5_O_7_	3.5	9.59 ± 0.19	89.02 ± 0.64	76.56 ± 1.22
18		Na_3_C_6_H_5_O_7_	5	8.1 ± 1.41	84.87 ± 1.32	71.43 ± 2.36

^a^A mixture of alkene (1 mmol), octanoic acid (1 mmol), cell (200 mg), H_2_O_2_ (30% aqueous solution, 7.5 mmol), 1−5 mmol buffer salts (Na_2_CO_3_, Na_2_HPO_4_, and Na_3_C_6_H_5_O_7_) in toluene (1.5 mL) was shaken at 180 rpm and 30 °C for different reaction time

^b^The value obtained at 30 min for pinene and 60 min for octene and styrene, respectively

^c^The value obtained at 6 h for pinene, 50 h for octene and 58 h for styrene, respectively

It was found that the three alkenes all had low conversions. The conversion of α-pinene was 26.13% and the yield was 6.52% without the addition of buffer salts to the reaction system. This is inconsistent with the literature reports that strong base and weak acid salts were used as acid-trapping reagents to stabilise the conversion and increase the yield and indicates that the trisodium citrate in this reaction system was not only used as an acid-trapping reagent.

**Fig. 2:**
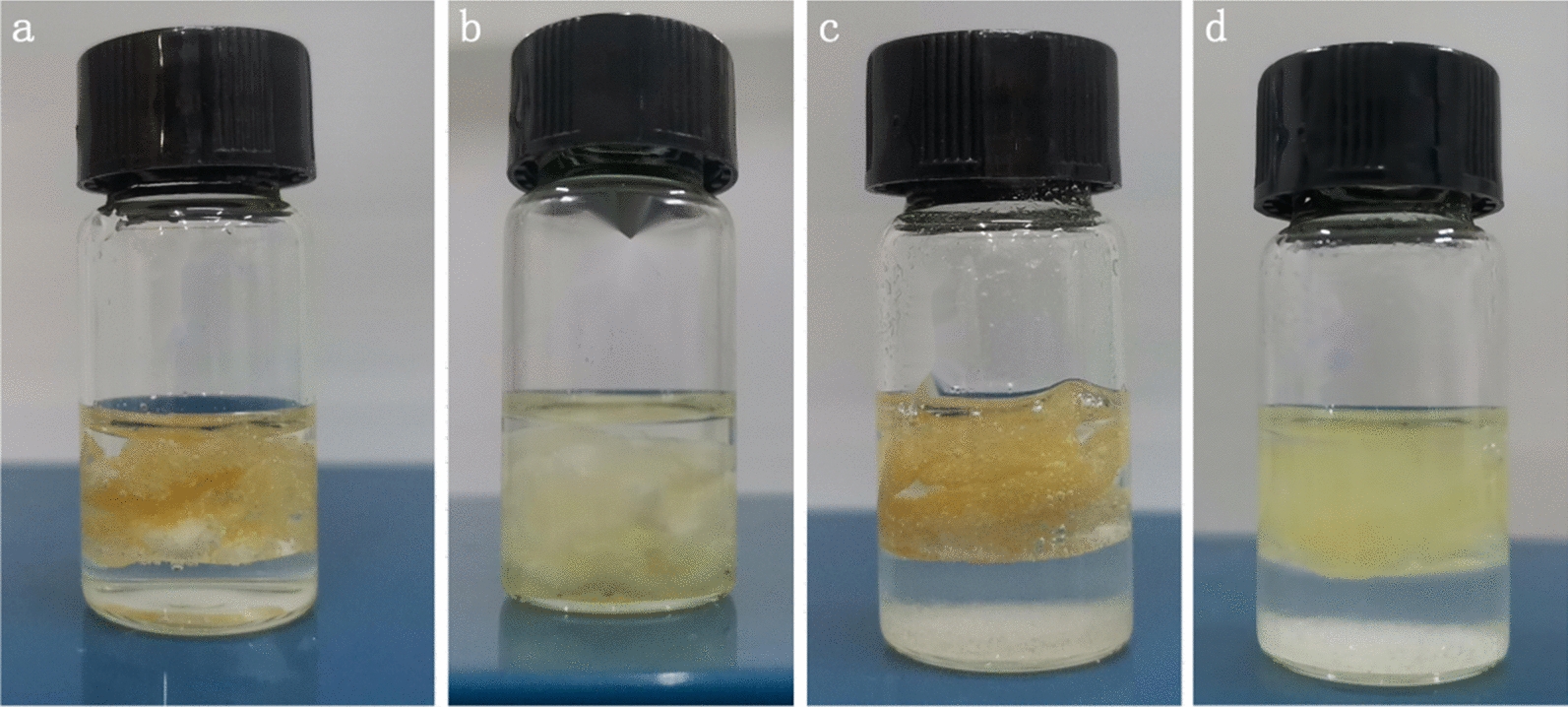
Whole-cell morphology in epoxidation systems with varied Na_3_C_6_H_5_O_7_. **a** Without the addition of Na_3_C_6_H_5_O_7_, reaction time 0 h. **b** Without the addition of Na_3_C_6_H_5_O_7_, reaction time 8 h. With the addition of 3.5 mmol Na_3_C_6_H_5_O_7_, reaction time **c** 0 h and **d** 8 h

*Rhizopus oryzae* whole-cell lipase required oil–water interface activation, suggesting that in solvent experiments, alkenes failed to undergo epoxidation with the organic solvents acetonitrile and isopropanol with logP values < 0.16 (Table 3). When the reaction system is free of buffer salts, the water in the reaction system penetrates the cells, causing the cells to sink into the aqueous phase (Fig. 2a, b), preventing activation at the oil–water interface and leading to the inactivation of intracellular lipase leakage. After adding various buffer salts, the aqueous phase will form a saturated salt solution. Equilibration of the cells with saturated salt solutions remains in the upper organic phase after 8 h of reaction (Fig. 2d) to give the best performance at 3.5 mmol Na_3_C_6_H_5_O_7_ (Table 6, entry 5, 11, 17).

**Fig. 3:**
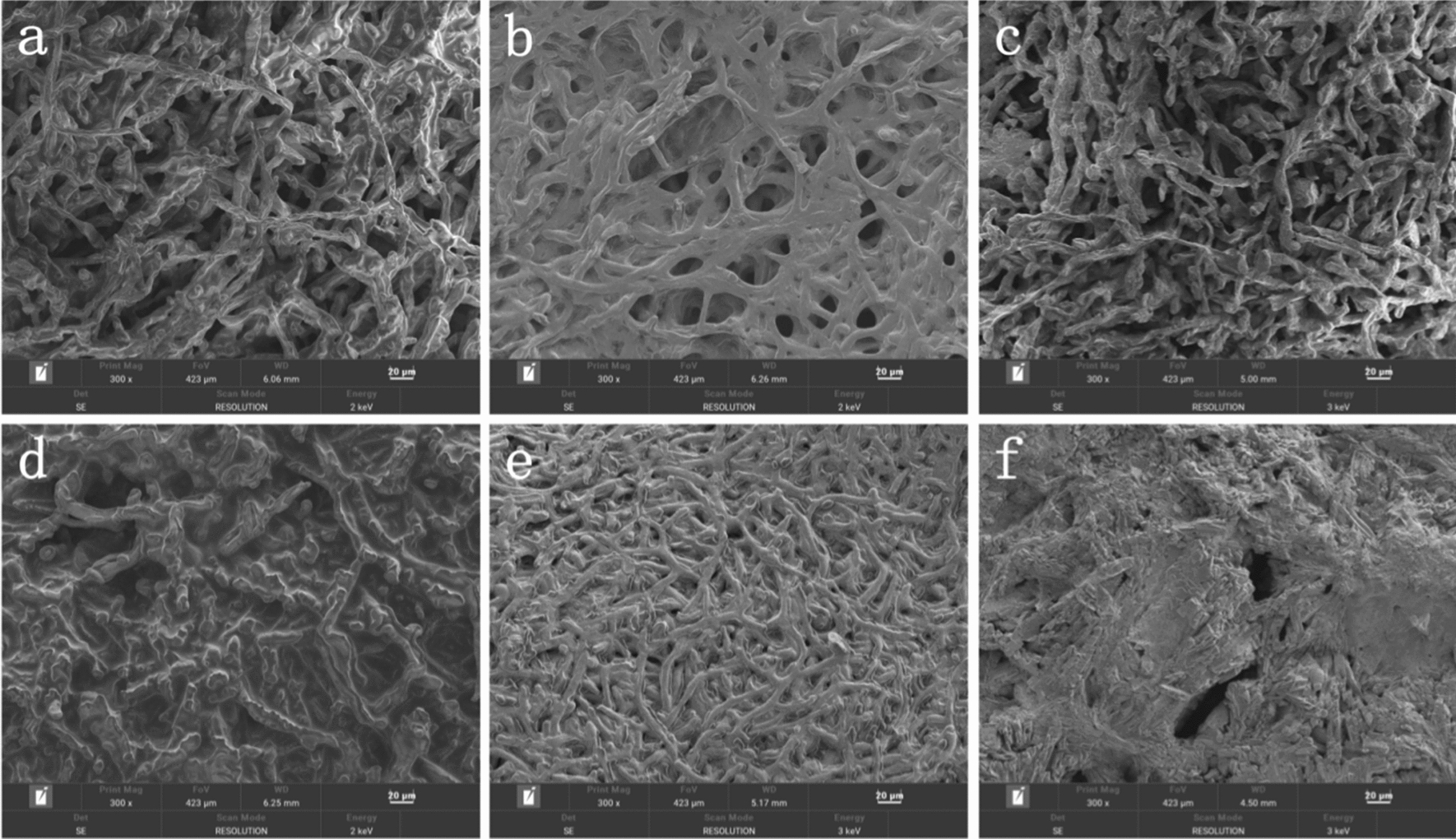
Scanning electron microscopy images of GA-crosslinked whole cells and untreated cells in epoxidation systems with varied buffer salts. **a** GA-crosslinked whole cells, reaction time 0 h. **b** GA-crosslinked whole-cells without the addition of buffer salts, reaction time 8 h. **c** GA-crosslinked whole-cells with 3.5 mmol Na_3_C_6_H_5_O_7_, reaction time 8 h. **d** Untreated cells, reaction time 0 h. **e** Untreated cells without the addition of buffer salts, reaction time 8 h. **f** Untreated cells with the addition of 3.5 mmol Na_3_C_6_H_5_O_7_, reaction time 8 h

The morphology of the crosslinked and non-crosslinked cells was observed by SEM in the presence or absence of buffer salts in the epoxidation reaction. It was found that the mycelium was thinner after crosslinking (Fig. 3a, d) and that the mycelium clumped together after reacting in an epoxidation system without added buffer salts by both the crosslinked and non-crosslinked cells (Fig. 3b, e). However, crosslinked cells in the epoxidation system with the addition of buffer salts maintained the sparse mycelium as before the reaction, while non-crosslinked cells changed the morphology of the mycelium. The intact and smooth cell walls were indeed broken down and agglomerated into solid clusters (Fig. 3c, f).

These results suggest that crosslinked cells show good catalytic activity possibly due to changes in cell surface properties. *Rhizopus oryzae* lipase is mainly located on the cell wall and cell membrane; the H_2_O_2_, substrate molecules, and product molecules can pass through the cell wall and can fully contact the lipase without causing the cell membrane to rupture in a saturated salt solution (Fig. 3a, c).

The concentration of H_2_O_2_ affected the efficiency of the epoxidation reaction and enzyme activity (Table 5). Comparing the concentration of H_2_O_2_ in the different phases of the reaction system at different amounts of trisodium citrate, it was found that different amounts of Na_3_C_6_H_5_O_7_ affected the concentration of H_2_O_2_ in the three phases and thus the epoxidation reaction.

**Fig. 4:**
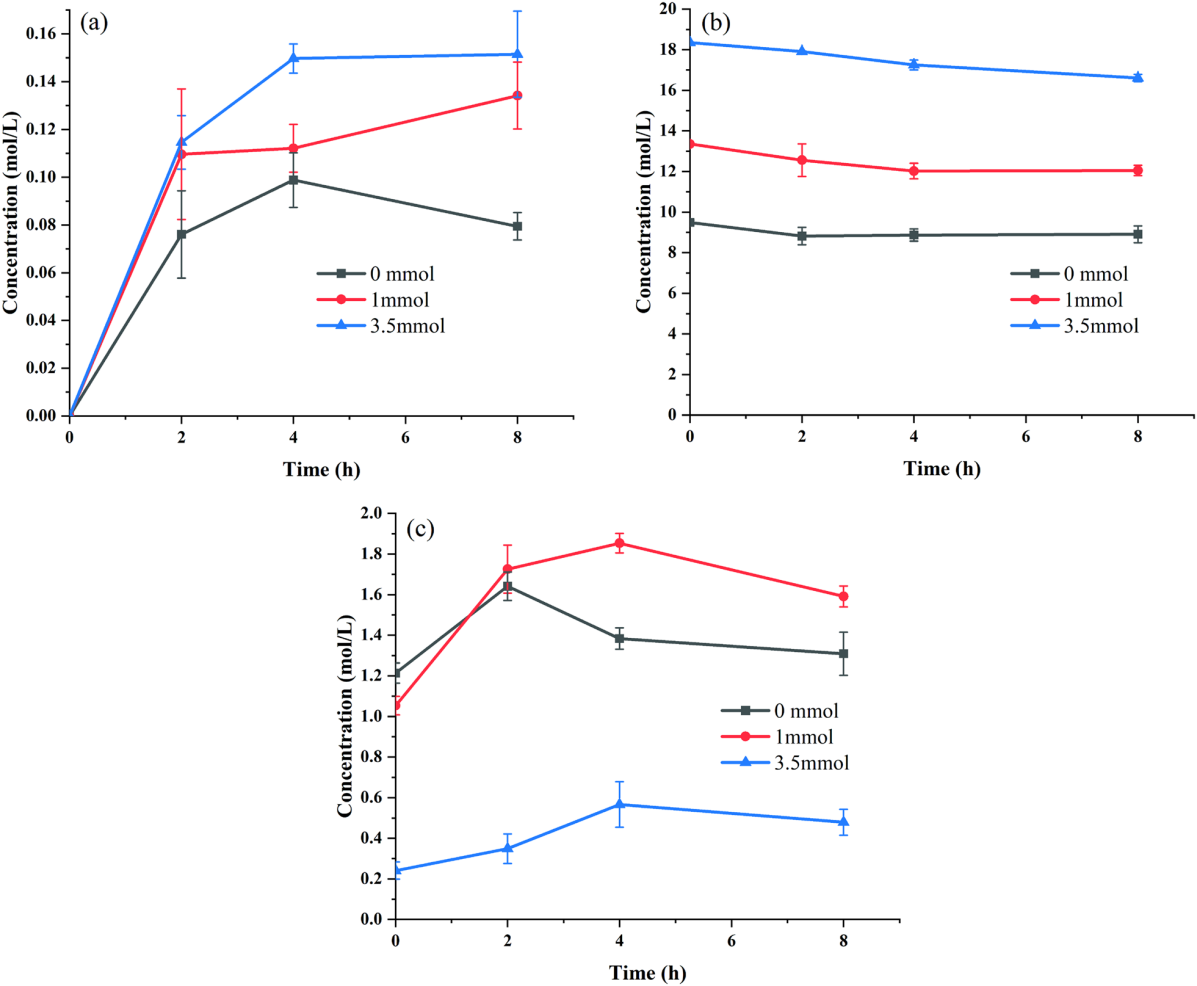
Variation in H_2_O_2_ concentration in whole-cell epoxidation with different Na_3_C_6_H_5_O_7_ concentrations at different phases [organic phase (**a**), aqueous phase (**b**), and solid phase (**c**)]

The H_2_O_2_ concentration in the organic phase gradually increased at all Na_3_C_6_H_5_O_7_ dosages. However, the H_2_O_2_ concentration in the organic phase could reach 0.15 mol/L by adding 3.5 mmol Na_3_C_6_H_5_O_7_, while the maximum concentration in the organic phase could only reach 0.098 mol/L without adding the buffer salt; the trend was decreasing (Fig. 4a). The H_2_O_2_ concentration in the organic phase was in the middle of the above two with the addition of 1 mmol Na_3_C_6_H_5_O_7_, indicating that Na_3_C_6_H_5_O_7_ could effectively increase the H_2_O_2_ concentration in the organic phase.

In the aqueous phase, as the amount of Na_3_C_6_H_5_O_7_ increased, the initial concentration of H_2_O_2_ increased, with the addition of 3.5 mmol Na_3_C_6_H_5_O_7_ the initial concentration of H_2_O_2_ in the aqueous phase was 18.346 mol/L. Without the addition of Na_3_C_6_H_5_O_7_, the initial concentration of H_2_O_2_ was 9.49 mol/L, i.e., 30% H_2_O_2_ original concentration, indicating that the addition of Na_3_C_6_H_5_O_7_ with 30% H_2_O_2_ solution formed a saturated salt solution, which in turn served to concentrate the H_2_O_2_ (Fig. 4b).

In the stationary phase, under osmotic pressure, a large amount of H_2_O_2_ solution enters the cells without the addition of Na_3_C_6_H_5_O_7_, leading to a rupture of the cell membrane and a decrease in the concentration of H_2_O_2_ in the cells (Fig. 4c, black line) according to Figs. 2b and 3b. The addition of 3.5 mmol Na_3_C_6_H_5_O_7_ resulted in only a certain amount of H_2_O_2_ solution entering the cells due to the formation of a saturated salt solution (Fig. 4c, blue line), which in turn reduces enzyme inactivation.

## Conclusion

We used GA-crosslinked *R. oryzae* whole cells as an enzyme for the epoxidation of a variety of alkenes. It was a three-phase system and the whole cells were located in the upper organic phase due to the addition of 3.5 mmol Na_3_C_6_H_5_O_7_. This whole-cell lipase needs to activate the interface to expose the active site and is only suitable for oxidation in a two-liquid phase. The Na_3_C_6_H_5_O_7_ not only acted as an acid-trapping reagent to eliminate the negative effect of the carboxylic acid on the alkene oxide but also formed a saturated salt solution with the aqueous phase, affecting the concentration of H_2_O_2_ in the three phases and thus the epoxidation reaction. This whole-cell epoxidation three-phase system could lead to biocatalysts becoming more resistant to the drastic reaction conditions originating from the use of H_2_O_2_ and peracids at high concentrations. Additionally, immobilisation can be used to solve the problem that cells are exposed to H_2_O_2_ in multiple cycles, which leads to cell damage and loss of enzyme activity. To summarise, whole-cell catalysis can eliminate enzyme purification processes and greatly reduce production costs.

## Supplementary Information

**Additional file 1**: **Figure S1. **Figure S1.GC chromatograms of alkenes and the corresponding epoxides standards. 1. α-pinene, 2. α-pinene oxide, 3. octane, 4. styrene, 5. octane oxide, 6. styrene oxide, 7. norbornene, 8. norbornene oxide, 9. α-methylstyrene, 10. α-methylstyrene oxide, 11. cyclohexene, 12. cyclohexene oxide, 13. 1-hexene, 14. 1-hexene oxide, 15. Toluene (Retention times: 4.3 min for α-pinene, 6.5 min for α-pinene oxide; 4.4 min for octane, 8.7 min for octane oxide; 6.5 min for styrene, 9.9 min for styrene oxide; 3.2 min for norbornene, 7.6 min for norbornene oxide; 8.3 min for α-methylstyrene, and 10.4 min for α-methylstyrene oxide; 2.7 min for cyclohexene, 5.7 min for cyclohexene oxide; 2.1 min for 1-hexene, 4.5 min for 1-hexene oxide, 4.1 min for solvent toluene). **Figure S2.** Evolution of the three model alkenes epoxidation reaction over time.

## Data Availability

The datasets supporting the conclusions of this article are included within the article and its additional file.
