# Supplementary material for: Glutaraldehyde-crosslinked Rhizopus oryzae whole cells show improved catalytic performance in alkene epoxidation
Source: Microb Cell Fact. 2023 Feb 22;22:33. doi: 10.1186/s12934-023-02026-0 (PMC9948446; doi:10.1186/s12934-023-02026-0)
Supplement: Supplementary file 1 — Additional file 1: Figure S1. Figure S1.GC chromatograms of alkenes and the corresponding epoxides standards. 1. α-pinene, 2. α-pinene oxide, 3. octane, 4. styrene, 5. octane oxide, 6. styrene oxide, 7. norbornene, 8. norbornene oxide, 9. α-methylstyrene, 10. α-methylstyrene oxide, 11. cyclohexene, 12. cyclohexene oxide, 13. 1-hexene, 14. 1-hexene oxide, 15. Toluene (Retention times: 4.3 min for α-pinene, 6.5 min for α-pinene oxide; 4.4 min for octane, 8.7 min for octane oxide; 6.5 min for styrene, 9.9 min for styrene oxide; 3.2 min for norbornene, 7.6 min for norbornene oxide; 8.3 min for α-methylstyrene, and 10.4 min for α-methylstyrene oxide; 2.7 min for cyclohexene, 5.7 min for cyclohexene oxide; 2.1 min for 1-hexene, 4.5 min for 1-hexene oxide, 4.1 min for solvent toluene). Figure S2. Evolution of the three model alkenes epoxidation reaction over time. [file 12934_2023_2026_MOESM1_ESM.docx]

**Additional file 1**

**Glutaraldehyde-crosslinked *Rhizopus oryzae* whole cells show improved catalytic performance in alkene epoxidation**

Lili Xu^1,2,3^ , Yimin Qin^3^, Yufeng Song^2^, Aixing Tang^3,4^ and Youyan Liu^3,4^*

1. Medical college, Guangxi University, Nanning, China 530004.

2. College of Marine Sciences, Beibu Gulf University, Qinzhou, China, 535011.

3. School of Chemistry and Chemical Engineering, Guangxi University, Nanning, China 530004.

4. Key Laboratory of Guangxi Biorefinery, Guangxi University, Nanning, China 530004.

*Corresponding author: Youyan Liu

E-mail address: liuyouyangx@hotmail.com

Figure S1.GC chromatograms of alkenes and the corresponding epoxides standards. 1. α-pinene, 2. α-pinene oxide, 3. octane, 4. styrene, 5. octane oxide, 6. styrene oxide, 7. norbornene, 8. norbornene oxide, 9. α-methylstyrene, 10. α-methylstyrene oxide, 11. cyclohexene, 12. cyclohexene oxide, 13. 1-hexene, 14. 1-hexene oxide, 15. Toluene (Retention times: 4.3 min for α-pinene, 6.5 min for α-pinene oxide; 4.4 min for octane, 8.7 min for octane oxide; 6.5 min for styrene, 9.9 min for styrene oxide; 3.2 min for norbornene, 7.6 min for norbornene oxide; 8.3 min for α-methylstyrene, and 10.4 min for α-methylstyrene oxide; 2.7 min for cyclohexene, 5.7 min for cyclohexene oxide; 2.1 min for 1-hexene, 4.5 min for 1-hexene oxide, 4.1 min for solvent toluene)

Figure S2. Evolution of the three model alkenes epoxidation reaction over time
